# Adsorption of Sodium Diclofenac in Functionalized Palygoskite Clays

**DOI:** 10.3390/ma15082708

**Published:** 2022-04-07

**Authors:** Matheus Urtiga Sousa, Alisson Mendes Rodrigues, Maria Eduarda Barbosa Araujo, Romualdo Rodrigues Menezes, Gelmires Araújo Neves, Hélio Lucena Lira

**Affiliations:** 1 Graduate Program in Materials Science and Engineering (PPG-CEMat), Federal University of Campina Grande, Av. Aprígio Veloso-882, Bodocongó, Campina Grande 58429-900, PB, Brazil; mariaeduardaba@hotmail.com; 2 Laboratory of Materials Technology (LTM), Department of Materials Engineering, Federal University of Campina Grande, Av. Aprígio Veloso-882, Bodocongó, Campina Grande 58429-900, PB, Brazil; alisson.mendes@professor.ufcg.edu.br (A.M.R.); romualdo.menezes@ufcg.edu.br (R.R.M.); gelmires.neves@ufcg.edu.br (G.A.N.); helio.lira@ufcg.edu.br (H.L.L.)

**Keywords:** palygorskite clay, adsorption, drug, design of experiment

## Abstract

The effects of acid and organo-functionalizations on the surface of Brazilian palygorskite clay was investigated, evaluating its potential in the adsorptive capacity of the drug sodium diclofenac present in wastewaters. The modifications on the clay structure were investigated by X-ray diffraction, X-ray fluorescence, thermogravimetric, differential thermal analysis, Fourier transform infrared spectroscopy, surface area by N_2_ adsorption (77.5 K) and Zeta potential. The experimental design was carried out to find the best conditions for the adsorption tests, in which concentration, mass and pH were significant. In the kinetic study, the pseudo-second-order model better described the adsorption process for acid and organo-functionalized samples. Such results indicate that the adsorption behavior probably occurs due to the phenomenon of chemisorption. Regarding the adsorption isotherms, the Langmuir model was the one that best adjusted both the experimental data of acid and the organo-functionalized samples, whose maximum adsorption capacity were 179.88 and 253.34 mg/g, respectively. This model also indicates that the sodium diclofenac is adsorbed to monolayers homogeneously through chemisorption. In general, the studied clays proved to be suitable adsorbents for the removal of sodium diclofenac.

## 1. Introduction

Among the extensive list of micro contaminants commonly detected in wastewaters, drugs stand out because, even in low concentrations, they have a high potential to cause adverse effects on fauna and flora [1,2]. Such contaminants have minor to moderate biodegradability, increasing their environmental prevalence and the consequent impact on aquatic organisms. There is strong evidence that drugs in wastewater can impact the environment and human health, especially children [3].

When excreted, contaminants such as sodium diclofenac (SD) are inevitably sent through the wastewater drainage systems, being conducted to the wastewater treatment plant (WWTP). However, in most cases, the WWTP effluents are subjected to conventional treatment processes that are generally ineffective in removing some drugs, resulting in their almost integral discharge in the receiving environment. Therefore, the reuse of these effluents can contaminate the final customer. Furthermore, the medicine’s packaging is another water contamination source since they are often discarded on the ground or sent to landfills, thus contaminating and directly influencing the locality’s environmental health and the water body users [4,5,6]. Many compounds frequently detected in natural waters and treated effluents belong to antibiotics, lipid regulators, beta-blockers, neuroactive and anti-inflammatory drugs [7,8,9].

Sodium diclofenac (C_14_H_10_Cl_2_NNaO_2_) is a white crystalline or slightly yellow solid with negligible water solubility, which acts as a competitive and irreversible inhibitor of prostaglandin synthetase. Its analgesic and anti-inflammatory activities are based on preventing araquinodate metabolite synthesis via inhibition of cyclooxygenase [10]. In humans, the SD toxicological effects are still not well understood; however, some studies show a relationship between SD founded in the environment with dysfunctions in the endocrine system, such as abnormalities in human sexual organs and low blood count [11,12,13].

Several removal processes and/or treatment of drugs from the wastewaters have been proposed. Among them are nanofiltration, photocatalysis, coagulation/flocculation, membrane filtration, electrochemical methods, and adsorption [14,15,16,17,18,19,20,21,22,23,24]. Adsorption is an excellent technique because it is easy to operate, has adsorbate selectivity, has a good efficiency in removing pollutants, and has adsorbent reusing potential [25,26]. At the moment, some adsorbents (such as carbon nanotubes, activated carbon), despite having high porosity and surface area, usually have increased production and regeneration costs, which can make them economically unfeasible, in addition to being from the burning of natural resources [27]. In this sense, it is essential to develop research to search for low-cost natural adsorbents with the same characteristics as the adsorbents above, such as clays. Nowadays, there has been an increase in research that monitors the removal of sodium diclofenac by adsorption on organo-functionalized bentonite [18,27,28,29,30,31,32,33] by lamellar morphology and great possibility of expansion. On the other hand, fibrous clays, porous texture considerable and relevant surface properties such as palygorskite are hardly reported, which can be a way to be explored and that deserves to be highlighted.

A palygorskite clay is a hydrated magnesium aluminum silicate with the theoretical formula of Si_8_Mg_8_O_20_ (OH)_2_ (H_2_O)_4_ · 4H_2_O [34] An isomorphic phenomenon is frequently observed, in which the central Mg^2+^ octahedral cations are often replaced by Al^3+^ or Fe^3+^, resulting in a dioctahedral or intermediate structure [35]. Such clay is extremely versatile have the following characteristics: ripiform fibrous morphology, porous texture, fine granulometry, high surface area, low toxicity and sorption capacity of different species, mainly cationic [36,37,38,39,40], it has an abundance in northeast Brazilian, 20 times cheaper than activated carbon [41] and different chemical-mineralogical characteristics, where one of the main applications is in the use in nanoformulations of sodium diclofenac carriers for controlled release [42,43,44,45,46], showing the great applicability of this type of clay. Therefore, studying the adsorption on such clays is relevant to scientific knowledge and technological advances. In general, palygorskite clays present a predominance of negative charges, suggesting low adsorption performance of anionic contaminants such as sodium diclofenac, in this sense it is necessary to perform functionalizations.

In recent years, there were relevant works involving the functionalization of clays by pillaring for adsorptive processes in waste water treatment [47,48]. While for fibrous clays, types to be highlighted include: thermal functionalization [49], acid functionalization [26,40] and by organo-functionalization [50,51]. Its application was restricted to sulfur adsorption in fuels, toluene, dyes, heavy metals and terpenic compounds, mainly by foreign palygorskites. As far as is known, no research approached in a comparative way involving different functionalizations in the structure of Brazilian palygorskite for application as an adsorbent for the drug sodium diclofenac, therefore, the objective of this work was to analyze the effects of acidic and organic functionalizations on the surface of native palygorskite clay from Northeast Brazil, evaluating its potential in the adsorptive capacity of the drug diclofenac sodium present in wastewater.

## 2. Materials and Methods

### 2.1. Raw Materials

The palygorskite clay used in this work was supplied by União Brasileira de Mineração S.A (UBM, Guadalupe, Piauí state, Brazil), presenting average diameters of 8.38 μm and Cation Exchange Capacity (CEC) 21.96 meq/100 g. The sulfuric acid (Química Moderna, MM: 98.08 g·mol^−1^, 98%) and the Praepagen WB^®^ (Clariant INC., São Paulo, São Paulo State, Brazil) were the reagents that used acid and organic functionalization, respectively. The Praepagen WB^®^ was made with distearyl dimethyl ammonium chloride (75% active substance whose solvent is water). The hydrochloric acid and ammonium hydroxide (VETEC, Brazil) were used for pH adjustment. The sodium diclofenac (Fagron, 98.5%, internal lot 17H10-B021-022127) was the adsorbate used for the adsorption studies.

### 2.2. Acid and Organic-Functionalization of Raw Palygorskite

An amount of 25 g of raw palygorskite were immersed in 250 mL of H_2_SO_4_ solution (6N) in a reflux system. The reflux system was kept under stirring and constant temperature (90 °C). After 2 h, the system was filtered and washed with distilled water under vacuum until pH = 7. After this, the material obtained was dried at 60 °C for 48 h, ground and sieved (0.074 mm) [52]. It was decided to work with this concentration due to the resistance to acid attack of this type of clay and the increase in surface area [53].

The organic functionalization was accomplished using the cationic surfactant (Praepagem WB^®^, distearyl dimethyl ammonium chloride). The experimental protocol used was based on the CEC [18]. First, the cationic surfactant was dissolved in distilled water (500 mL). Then, 50 g of raw palygorskite clay was added to the solution under constant stirring (2000 RPM) for 1 h at room temperature. The system was filtered and washed until chloride ions were not detected by the 0.1 mol·L^−1^ silver nitrate solution. The filtrate obtained was dried at 60 °C for 48 h, ground, and sieved through a 200 mesh (0.074 mm).

### 2.3. Characterizations

X-ray diffraction experiments (XRD-6000, Shimadzu, Kyoto, Japan) were performed using CuK radiation (λ = 0.15406 nm), operated at 40 kV and 30 mA, goniometer speed of 2°/min, angular step of 0.02, and a counting time of 0.6 s. The chemical analysis was determined by X-ray fluorescence spectrometry (EDX 720-Shimadzu, Kyoto, Japan). Thermogravimetry (TG) and differential thermal analysis (DTA) were performed under an air atmosphere, with a heating rate of 12.5 °C/min (DTG-60H-Shimadzu, Kyoto, Japan) in a platinum crucible from room temperature to 1000 °C. Fourier-transform infrared spectroscopy (FTIR) was recorded in the spectral range from 4000 cm^−1^ to 400 cm^−1^, 32 scans with a resolution of 4 cm^−1^ by attenuated total reflectance (ATR) accessory in diffuse reflectance mode at room temperature (Perkin Elmer, Spectrum 400—Waltham, MA, USA). The surface area quantification was carried out using the Brunauer–Emmett–Teller (BET) method utilizing N_2_ adsorption (Nova 1200e Quantachrome Autosorb iQ, Anton Paar, Graz, Austria) [54,55]. Zeta potential was measured using a Zetasizer Nano Zs (Malvern Instruments, Malvern, England) for isoelectric titration through pH titration. The pH of the solutions was adjusted with 0.100 mol·L^−1^ NH_4_OH or 0.500 mol·L^−1^ HNO_3_.

### 2.4. Experimental Design 

An experimental design was carried out to evaluate the influence of independent experimental variables on the adsorptive capacity of acid and organo-functionalized samples. Such procedure was accomplished to determine the best experimental conditions. The independent variables used in this work are listed in Table 1 with decoded and real values. This methodology uses statistical tools to evaluate a system’s efficiency through the variables and their influence on the response obtained. Therefore, it consists of organizing the input variables into an experiment series, varying them from high (upper) to low (lower) levels [56].

Based on the experimental design conditions presented in Table 1, the samples were placed in a refrigerated shaker incubator-NT 735 (Nova Técnica, São Pualo, Brazil), with a stirring speed of 200 rpm and room temperature (25 °C). The initial pH of the solution was adjusted by adding 0.1 HCl or NH_4_OH. 10 mL were removed from the samples, centrifuged for 10 min at 3600 rpm, and then analyzed using UV-VIS spectroscopy (model UV-1800, Shimadzu, Kyoto, Japan) at 292.16 nm. The experimental design was performed in triplicate at the central point, resulting in 38 experiments. The responses used to determine the process efficiency were the adsorptive capacity $Q_{\mathrm{ef}}$ (mg/g), Equation (1), and the SD ions adsorption removed percentage (% Rem), Equation (2):

(1) $$Q_{\mathrm{ef}}=\frac{V\left(C_{i}-C_{e}\right)}{M}$$

(2) $$\%\mathrm{Rem}=\left(\frac{C_{i}-C_{e}}{C_{i}}\right)\times 100$$

where C_i_ (mg/L) is the initial SD concentration; C_e_ (mg/L) is the concentration of the SD in equilibrium; M (g) is the adsorbent mass (clay); V (L) is the solution volume of SD.

### 2.5. Kinetics and Equilibrium Models 

For the kinetic study, the best adsorption tests experimental conditions were evaluated, varying only the time. The experimental data will be fitted to the pseudo-first-order (Equation (3)) [57], pseudo-second-order (Equation (4)) [58], and Elovich models (Equation (5)) [59]:

(3) $$Q_{tfo}=Q_{ef}\left(1-e^{-k1\text{.}t}\right)$$

(4) $$Q_{tso}=\frac{k_{2\;}Q_{ef\;}^{2}t}{1+k_{2\;}Q_{ef}t}$$

(5) $$Q_{tel}=\frac{1}{\beta }\ln\left(1+\alpha \beta t\right)$$

where the $Q_{tfo}\;,\;Q_{tso},\;Q_{tel}$ are the adsorption capacities at time t (min) for the kinetic models of pseudo-first-order, pseudo-second-order and Elovich, respectively. $Q_{\mathrm{ef}}$ (mg·g^−1^) is the adsorptive capacity at equilibrium. In Equation (5), α (mg·g^−1^·min^−1^) is initial adsorptive rates, and β (g·mg^−1^) is the Elovich desorption constant. Both are related to the extension of the surface coverage and the activation energy involved in the chemisorption.

The adsorption isotherms were obtained using the best experimental conditions of the adsorption tests, which will be varied the SD concentration. In the sequence, the experimental data were adjusted by Langmuir (Equation (6)) [60,61], Freundlich (Equation (7)) [62], and Temkin isothermal models (Equation (8)) [63]:

(6) $$Q_{eL}=\frac{Q_{máx}.{\;K}_{L}.C_{e}}{1+K_{L}.C_{e}}$$

(7) $$Q_{\mathrm{ef}}=K_{f}.C_{e}^{1/n}$$

(8) $$Q_{et}=\frac{RT}{\beta_{t}}\ln\left(\alpha_{t}.C_{e}\right)$$

where, in the Langmuir model, $Q_{eL}$ (mg·g^−1^) is the SD adsorbed amount on the solid/liquid interface, $Q_{máx}$ (mg·g^−1^) is the maximum adsorptive capacity to form a monolayer on the palygorskite surface, $C_{e}$ (mg·L^−1^) is the equilibrium concentration of the SD and $K_{L}$ is the Langmuir constant. In the Freundlich model, $Q_{\mathrm{ef}}$ (mg·g^−1^) is the SD adsorbed amount on the solid/liquid interface, $C_{e}$ (mg·L^−1^) is the equilibrium concentration of the SD, $K_{f}$ (mg·g^−1^).(mg·L^−1^)^−1/n^ and n are Freundlich’s empirical constant, being a factor that is related to the adsorption capacity and intensity. For the Temkin model, $Q_{et}$ (mg·g^−1^) is the SD adsorbed amount on the solid/liquid interface, C_e_ (mg·L^−1^) is the equilibrium concentration of the SD, $\beta_{t}$ is the constant related to the adsorption energy (J mol^−1^), $\alpha_{t}$ (L mg^−1^) is the Temkin isotherm constant, R is the gas constant (8.314 J mol^−1^K) and T is the temperature (K).

All the experiments were performed in duplicate.

## 3. Results and Discussion

### 3.1. Characterization of the Raw and Functionalized Palygorskite

#### 3.1.1. X-ray Diffraction

**Figure 1:**
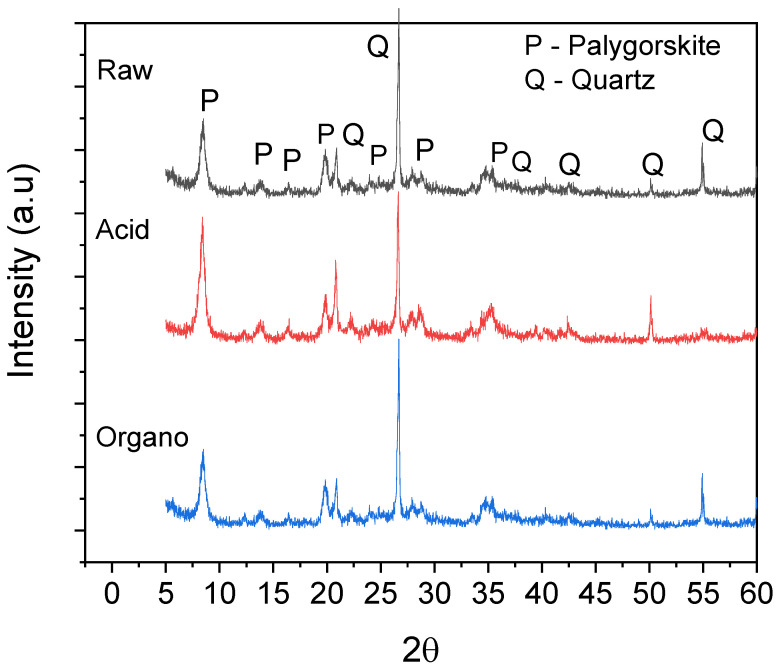
X-ray diffraction (XRD) pattern of raw and functionalized palygorskite.

X-ray diffraction (XRD) patterns of raw and functionalized samples are illustrated in Figure 1. Palygorskite (ICCD 21-0958) and quartz (ICCD 46-1045) phases were identified in the raw and functionalized samples, as observed in other studies [64,65]. It is observed that the structure of the raw palygorskite has not been substantially modified by acid functionalization; however, due to the stability of quartz in an acidic medium [66], a slight increase in the quartz peak (22.5°and 50°) and a reduction in the quartz peak at 55°, causing an amorphization which can be linked to a reduction in the crystallinity of the structure was detected in the acid-functionalized sample. In the organo-functionalized sample, no apparent change in XRD patterns was observed in raw palygorskite.

#### 3.1.2. X-ray Fluorescence Chemical Composition

The chemical composition (Table 2) measured from the raw and functionalized samples identified the SiO_2_, Al_2_O_3_, Fe_2_O_3_, MgO as majority contents. Such results agree with previous works, which also investigated similar palygorskite [67,68]. It is known that the palygorskite clay can be classified into three types: Type I has similar Al and Mg contents and negligible isomorphic substitutions (d200 < 0.635 nm), type II is rich in Al contents and has a dioctahedral character, and this is the most commonly found type. Type III has a trioctahedral character, mostly magnesian. The palygorskite investigated in this work has a d200 equal to 0.641 nm; therefore, it belongs to type II [36], but there is Brazilian palygorskite of a trioctahedral character (type III) [40].

The acid attack breaks the palygorskite clay fibers causing partial destruction of the structure and leaching of octahedral ions located at the edges, particularly those ions Al^3+^, Fe^3+^ or Fe^2+^and Mg^2+^ [69]. In contrast, the silica located in the tetrahedral layer of the palygorskite structure remains intact. It was also evident, through the X-ray fluorescence chemical composition, that the reduction of these cations located in the octahedral structure follows the order Al^3+^ > Fe^3+^ > Mg^2+^, which can be explained by the position occupied by the cations of the octahedral layer of the palygorskite structure, being first attacked by the proton (H^+^) from the acid the cations located on the octahedral border and in a row those that occupy the adjacencies [26].

Also, in Table 2, the organo-functionalized sample showed a chemical composition similar to the raw sample, except for the chemical component Cl, which has origin from the addition of the cationic surfactant that accommodated the raw palygorskite’s surface.

#### 3.1.3. Thermal Behavior

**Figure 2:**
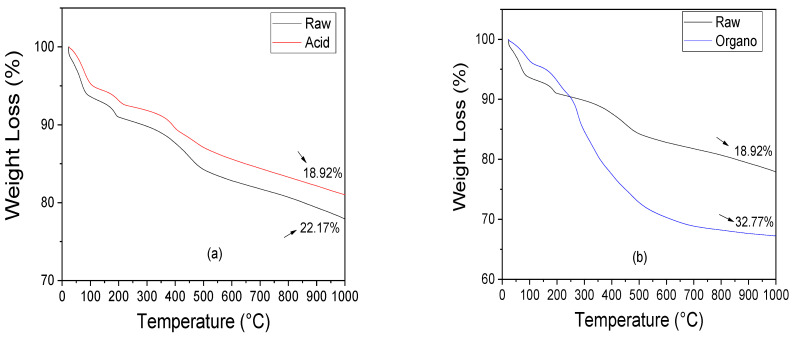

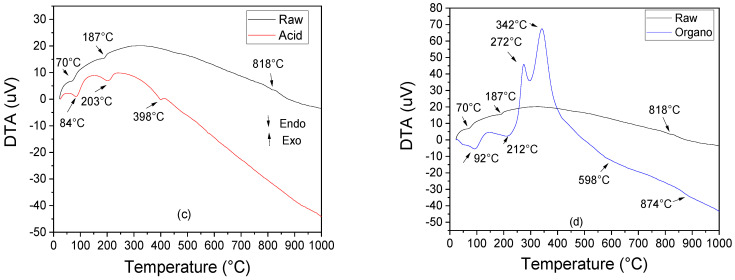
Compare TG and DTA curves of raw sample with the (**a**,**c**) acid functionalized, and (**b**,**d**) organo-functionalized samples.

TG and DTA curves of raw and functionalized samples are shown in Figure 2. Four mass loss stages were identified in the TG curves of the raw sample (Figure 2a). In the first thermal stage (24.62–84 °C), a mass loss equal to 6.03% was observed and is attributed to the evaporation of the water physically adsorbed on the sample surface [70]. A mass loss equal to 3.14% was detected in the second thermal stage (84–195 °C) and is attributed to zeolitic water loss located in the palygorskite channels [71]. In the third thermal stage (195–498 °C), a mass loss equal to 6.71% was detected. Such mass loss is attributed to the coordinated water loss and the aluminol silanol and groups condensation [36]. In the fourth thermal stage (498–1000 °C), there was a mass loss equal to 6.29%. The TG curves of the acid-functionalized sample have a lower total mass loss than the raw palygorskite, due to the hydrolysis process providing a lower release of physiosorbed water molecules from the structure, corroborating the work of [40].

Three mass loss stages were identified in the organo-functionalized sample (Figure 2b). In the first thermal stage (25 to 110 °C), a mass loss equal to 4% was observed and is attributed to free and adsorbed water on the external palygorskite surface. In the second thermal stage (110–269 °C), a mass loss equal to 7.23% is attributed to the surfactant decomposition and water molecules coordinating the central cations in the octahedron at the edge of the octahedron ribbon. In the third thermal stage (269 to 505 °C), there was a loss of mass equal to 16.23%, attributed to the combustion and decomposition of the surfactant. In the fourth thermal stage (505 to 1000 °C), a mass loss equal to 5.31% is attributed to dehydroxylation of the crystalline structure. Moreover, by using TG curves, it is possible to quantify the percentage of incorporated cationic surfactant. In this work, it was 46.09%.

The DTA curves of the raw and functionalized samples corroborate the results of the TG curves. It was observed that the acid-functionalized sample (Figure 2c) presented thermal stages similar to the raw sample. The exception was an endothermic peak at 398 °C, attributed to the SO_3_ anion loss referring to sulfuric acid. It was also observed that the organo-functionalized sample (Figure 2d) showed exothermic peaks at 272 °C and 342 °C, attributed to the combustion and decomposition of the surfactant, respectively, corroborating with studies of [72].

#### 3.1.4. Specific Surface Area by N_2_ adsorption (BET)

**Figure 3:**
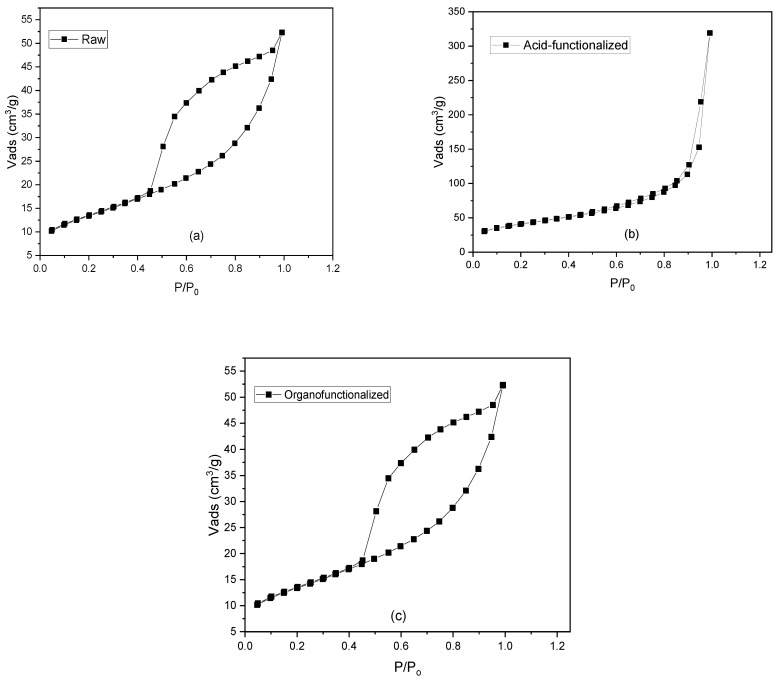
N_2_ Adsorption isotherms (77.5K) of raw (**a**), acid functionalized (**b**) and organo-functionalized samples (**c**).

The N_2_ adsorption isotherms of the raw and functionalized samples are illustrated in Figure 3 as well as experimental data of specific surface area (BET) and average pore diameter are summarized in Table 3. The BET area and average pore diameter in the acid-functionalized sample increased, with a probability of an increase in the active adsorption sites, due to the removal of impurities (oxides) by leaching of octahedral cations [40]. On the other hand, the organo-functionalized sample showed a decrease in surface area values, which may infer that the surfactant loading has an influence the surface characteristic, where a similar result was reported by [72]. The raw sample presents values of the specific surface similar to literature [73].

#### 3.1.5. Zeta Potential

**Figure 4:**
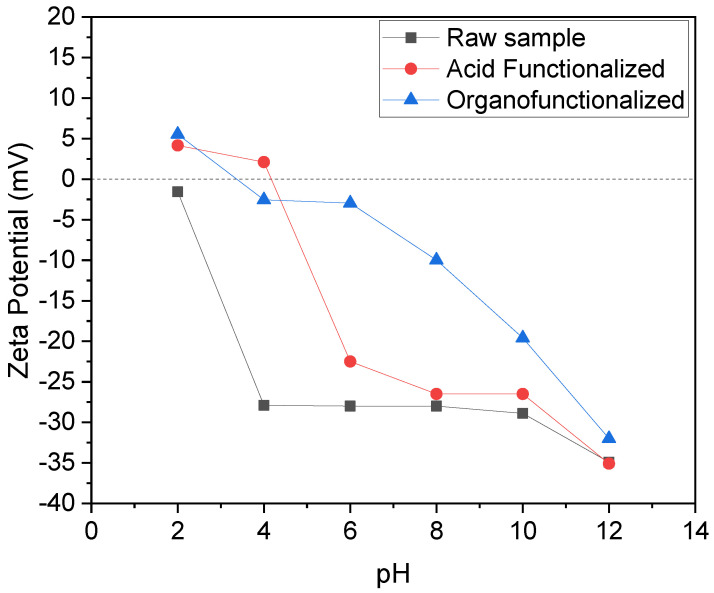
Zeta potential measurements for raw sample and functionalized clays.

It is observed that Zeta potential measurements by varying the pH (Figure 4) suggested that the raw sample has a negative charge in all pH ranges, but it was possible to observe the isoelectric point of acidic and organo-functionalized palygorskite at pH = 4.14 and 3.38, respectively. Therefore, at pHs lower than these values, the surface tends to be positively charged and on the other hand, when pH is higher than these values, the surface tends to be negatively charged. After conducting preliminary tests, it was observed that the raw sample did not show adsorptive efficiency for the SD drug, probably due to the predominance of negative charges, which can cause electrostatic repulsion. However, for the functionalized samples, a considerable adsorptive efficiency was obtained, probably due to the increase in in silanols from the acid and consequent electrostatic attraction or surface phenomenon (increase in surface area) as well as non-electrostatic attractions (adsorbate-adsorbent organic interactions). In this sense, the study was subsequently restricted to functionalized samples only.

### 3.2. Influence of pH

**Figure 5:**
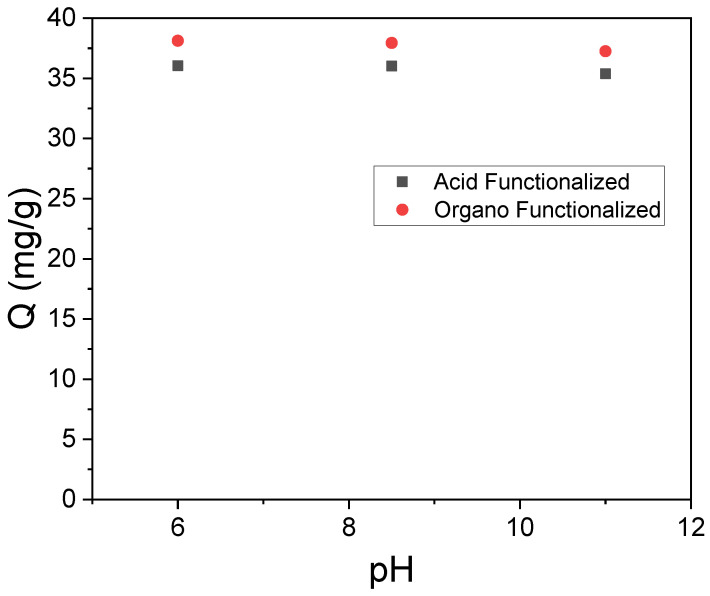
Effect of pH on the adsorption of SD by acid and organo-functionalized clays (m = 0.05 g, T = 25 °C and C_i_ = 50 mg L^−1^).

The effect of pH in the adsorptive process has often been evaluated according to the different behaviors presented by drugs and clays in aqueous media, as it can affect at the same time the surface charge of the adsorbent, the ionization degree of the functional groups of adsorbate and the adsorption mechanism. This parameter was analyzed to investigate the influence of the pH solution on adsorption; the pH range of 6–11 (Figure 5) was used. It was observed that the adsorption of drugs by the functionalized clays depended slightly on the pH. Still, the amount adsorbed as a function of pH in aqueous solutions was practically constant, with minimal variations (standard error: 0.118 and 0.143 for acidic and organo-functionalized samples, respectively).

The SD speciation diagram shows that the anionic form is predominant (pKa 4.1) [18], and the adsorbed amount slightly decreased with the increase of pH related to the electrostatic repulsion of anionic SD and functionalized clays. Suggesting that non-electrostatic interactions contributed to the adsorption mechanism.

### 3.3. Experimental Design of the Factorial Type of Functionalized Palygorskite Clay

**Figure 6:**
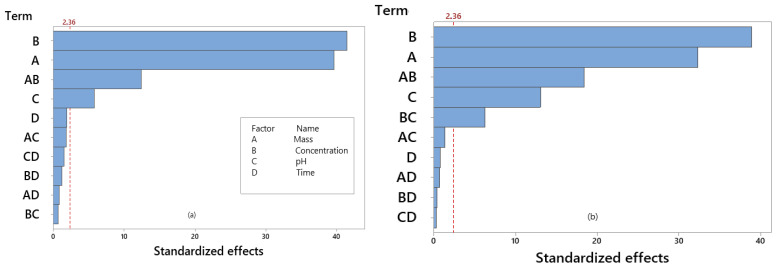
Pareto chart—Influence of the independent variables on the Q_t_ response variable of the functionalized palygorskite clay (**a**) acid and (**b**) organic (bars beyond the dashed line are statistically significant at the 95% confidence level).

**Figure 7:**
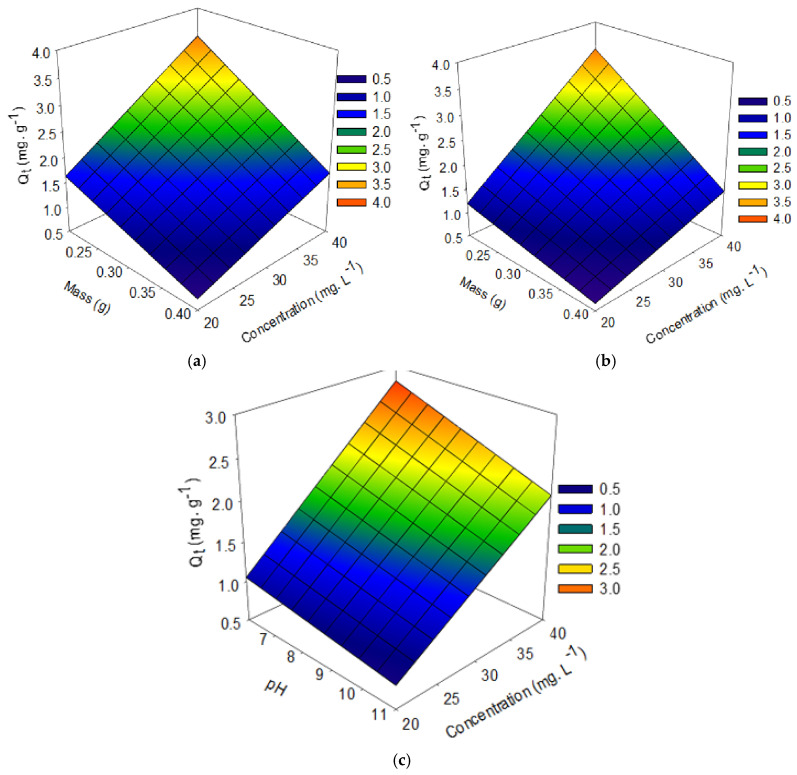
Contour chart for the Qt variable concerning to the interaction of the concentration of SD and mass variables of the acid-functionalized sample (**a**), organo-functionalized (**b**), concerning to interation of the pH (solution) and SD concentration in the organo-functionalized (**c**).

The results of the adsorption process obtained by statistical analysis can be seen in Table 4 (factorial experimental design), Table 5 and Table 6 (ANOVA and coefficient of determination of the factorial design), and Table 7 (quantification of effects) as well as illustrated in Figure 6 (Pareto chart—Influence of effects of the independent variables) and Figure 7 (Contour chart).

From the results of the factorial design (Table 4), it was observed that the best experimental conditions were 3 (m = 0.02 g, 40 mg/L, pH = 6, time = 4 h) for both functionalized clays. Researchers evaluated the performance of the acid-functionalized palygorskite at different toluene adsorption concentrations. The performance of the process suggested that the higher the acid concentration, the greater the adsorption capacity of the toluene [26]. From the application viewpoint, acid-modified adsorbents can interact with organic and inorganic compounds in an aqueous solution through the formation of complexes. Acid-functionalization creates Brönsted acid sites (positive nature) on the clay surface generated by the cations exchange in the interlamellar layers for acid-derived protons [74]. Another effect that also occurs is removing the ions from the octahedral layers and increasing the surface area of the clay mineral [75]. These active sites tend to attract the molecules of sodium diclofenac.

Still in Table 4, the organo-functionalized clay’s performance was attributed to better compatibility with organic pollutants, such as the drug under study, through its hydrophobic nature and the presence of new adsorption sites [9,65]. These behaviors are expected since the chemical modification favors the increase of the active sites on the adsorbent surface, favoring the adsorption phenomenon.

Table 5 and Table 6 show the analysis of variance of the experimental design for $Q_{t}$ obtained with both adsorbents (functionalized samples). It was observed, in the variance analysis, that the regression models obtained excellent adjustment to the experimental data, presenting very expressive determination coefficients, in which the models can explain more than 99% of the variations in the answers. According to [25], a mathematical model can be considered statistically significant if F_cal_ > F_tab_ and predictive if this relationship is greater than 10. So, for the variable Q_t_ with the samples investigated, the model can be considered statistically significant at the 0.05 confidence interval.

In the Pareto Charts, shown in Figure 6, the influences of the independent variables (mass, concentration, pH and time) on the response variable (Q_t_) are presented for the experimental designs accomplished. In the Pareto Charts, the bars of the factors that graphically exceed the significance line (*p* = 0.05) exercise a statistically significant influence on the results, being classified in decreasing order of importance. It is observed that the solution concentration, adsorbent mass, pH, as well as binomials mass and concentration are statistically significant at the confidence interval of 0.05 for the Q_t_ response with the acid-functionalized sample. While for the organo-functionalized sample, the binomial concentration and pH were statistically significant at the confidence interval of 0.05.

After analyzing the standardized effects on the Pareto charts, the quantification of the effects of the independent variables on the Qt response variable of the factorial design was performed for (a) acid and (b) organo-functionalized palygorskite clay. As shown in Table 7, the adsorbent mass variable negatively affected Q_t_ responses, indicating that the amount adsorbed was more significant under the lower level. The variable concentration of the solution had a positive effect showing that, with increasing drug concentration in the solution, availability increased and this condition facilitates the adsorption process. The pH variable had a small negative effect, indicating that the adsorption process tends to reduce with an increase in pH. Regarding the mass and concentration binomial, they had a negative effect, inferring that they are inversely proportional quantities. At the lowest mass and highest concentration level, the adsorption process is favored for both functionalizations. The binomial concentration and pH (statistically significant for the organo-functionalized sample) had a negative effect, which can infer that they are inversely proportional quantities, that at the highest concentration level and the lowest pH level, the process of adsorption is favored. This work shows that the adsorbed amount is more than double under conditions of higher initial drug concentration, under the same operating conditions (pH, contact time and mass), both in the acid-functionalized sample and also in the organo-functionalized sample.

Researchers evaluated sodium diclofenac adsorption in another type of organo-functionalized clay in a static system. They concluded that the increase in the solution concentration had a positive effect on the adsorptive capacity [33].

Other researchers monitored sodium diclofenac adsorption by organo-functionalized alkyl pyridinium bentonites in a static system. They concluded that the increase in the solution concentration, reducing the mass (up to 50 mg), regulating the system to slightly acidic pH and a contact time of up to 120 min favored the adsorptive process of the drug under study [18].

It was observed, in the contour graphs indicated in Figure 7, that to the independent variables mass and concentration, it can be inferred that the higher the initial concentration of the drug and the lower the clay mass, the greater the value adsorbed by the material for both functionalizations (a) and (b).

Regarding the independent variables concentration and pH (statistically significant for an organo-functionalized sample), it can be inferred that at the highest concentration level and lowest pH level, the higher the value adsorbed by the material for this type of functionalization (c), corroborating the best experimental condition obtained in the planning (C = 40 mg·L^−1^, m = 0.2 g of adsorbent mass and pH 6), obtaining an adsorption capacity equal to 3.768 mg·g^−1^ (functionalized acid), which corresponds to a removal of 94.2% and 3.42 mg·g^−1^ (organo-functionalized), which corresponds to a removal of 85.50%, as can be seen in Table 4.

### 3.4. Adsorption Kinetics

Adsorption kinetics studies of SD on the functionalized samples were performed using the masses (0.05 g), concentration (50 mg/L), pH = 6, steering of 200 RPM at room temperature. The contact time range investigated was 5 until 240 min. The pH was controlled during the adsorption kinetics experiments, as the pH can vary during the adsorption process, influencing the adsorption capacity of the drug under study. For this, it was measured before and after the processes, and it was found that they remained practically constant.

**Figure 8:**
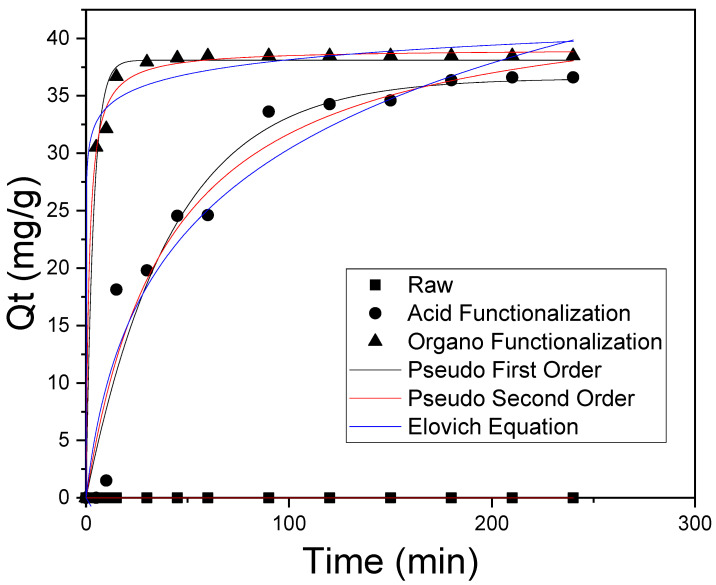
Representation of the fit for raw and functionalized samples to the kinetic model of pseudo-first-order, pseudo-second-order and Elovich Equation (m = 0.05 g, C = 50 mg·L^−1^, pH = 6, time = 4 h at 25 °C with 200 rpm rotation).

It can be seen from Figure 8 that the adsorption kinetics is slightly slow for the acid functionalized clay, reaching equilibrium in 150 min; on the other hand, for the organo-functionalized clay, it occurred in the first 15 min. This same Figure 8, Table 8 and Table 9 (Kinetic parameters and ANOVA) show the results obtained from SD adsorption kinetic experimental data on raw and functionalized clays from the non-linear fit to pseudo-first-order, pseudo-second-order and Elovich models.

The best mathematical adjustment was obtained from the pseudo-second-order model for both functionalized clays. The analysis of the best mathematical adjustment was performed in agreement with the R^2^ values (close to 1) and the minor difference between q_exp_ e q_teor_. Based on these kinetic models, it is possible to infer that the SD-adsorption in the functionalized sample occurs mainly by chemisorption [76,77,78,79,80,81], in a way that the molecules bind by chemical bonds, thus occurring the participation of valence forces or electron exchange between the adsorbent and the drug SD.

### 3.5. Adsorption Isotherms

The adsorption isotherms are important in determining the parameters related to the balance of the process. Such parameters describe how the adsorbate interacts with the adsorbent and, thus, predicts the maximum adsorption capacity of the adsorbent [82]. In this sense, the adsorption isotherm test of the drug was performed by evaluating SD concentrations, varying between 5–400 mg·L^−1^ in a 50 mL solution containing 0.05 g of functionalized clay, pH = 6, time of 4 h and stirring at 200 RPM, through the evidence of factorial design. The pH was controlled during the adsorption isotherm experiments, where it was measured before and after the processes, which remained practically constant.

The isotherms for the functionalized samples initially showed an increase in the adsorbed amount (Qef) with the increase in the concentration of the drug, corroborating the experimental design, which reached equilibrium at about 300 mg/L for the functionalized clays. Except for the raw one, palygorskite was insignificant as it did not show SD drug adsorption efficiency under the same conditions, probably due to the electrostatic repulsion between the negative charge on the clay surface and the negative nature of the SD. This result for functionalized clays is due to the increase in surface area (acid functionalization) suggesting numerous active sites available on the surface of the adsorbent in the early stages, as well as the strong non-electrostatic interaction (organo functionalization) between the surface of the palygorskite and the SD anionic.

**Figure 9:**
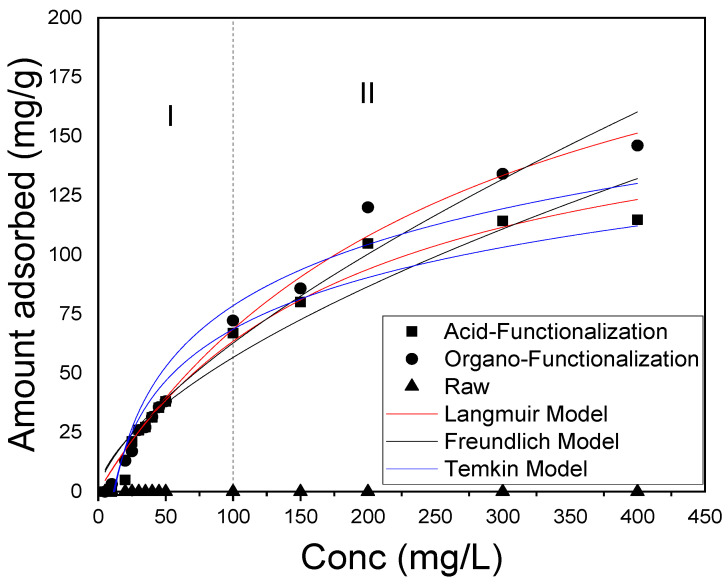
Representation of fit for raw and functionalized samples to the Langmuir isotherm models; Freundlich and Temkin (m = 0.05 g, pH = 6, time = 4 h at 25 °C with 200 rpm rotation).

The experimental data were fitted to the Langmuir, Freundlich, and Temkin isotherm models for the functionalized clays and compared to the raw sample, which had no adsorptive efficiency (Figure 9). This figure can be divided into two regions, where region I is included in the 5 mg/L and 50 mg/L SD rate. In this region, both the functionalized acid clay and the organo-functionalized clay adsorbed practically the same amount of the drug SD. As for region II, composed of the rates of 100 mg/L and 400 mg/L, it was noted that for the organo-functionalized clay, the Qef values were higher than that of the acid functionalized clay, with Qmax of 253.34 mg/g and 179.88 mg/g, respectively. The difference in the amount adsorbed between the functionalized clays can be attributed to the fact that the non-electrostatic interactions in the organo-functionalized clay are more intense than the surface phenomenon of the acid-functionalized clay.

Table 10 and Table 11 show that the isothermal and ANOVA parameters of the SD drug adsorption experimental data. It is observed that both functionalized samples had the best fit for the Langmuir isotherm model. Based on this model, it is possible to infer that the adsorption of adsorbate molecules occurs at sites with equivalent ionization energies present in the adsorbents. The drug’s molecules do not interact with each other and only a monolayer is formed in the adsorbent. The process that governs it is chemosorption, corroborating the kinetic model of pseudo-second order [36,40].

### 3.6. Characterization after Adsorption by FTIR

**Figure 10:**
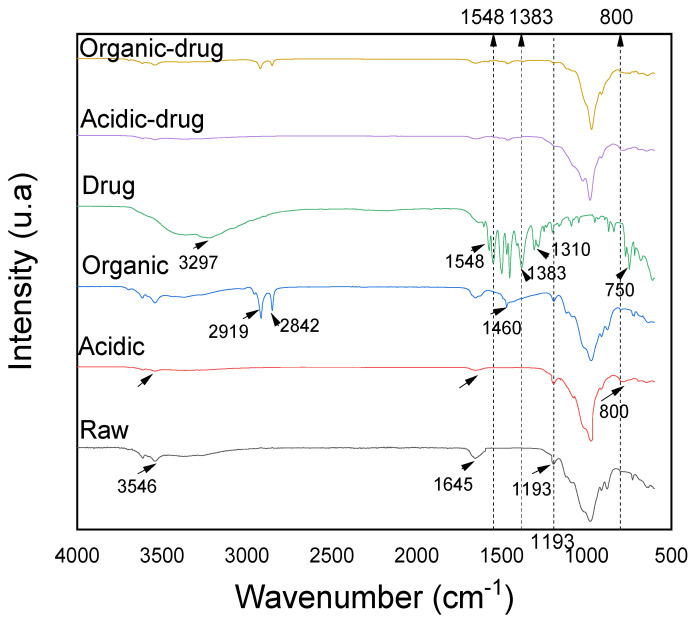
FTIR spectra of the raw and functionalized samples before and after the adsorption of the sodium diclofenac.

FTIR spectra data of functionalized samples are shown in Figure 10. In the raw sample was observed a band at 3546 cm^−1^ attributed to 2Al_2_-OH stretching vibration [83], band 1645 cm^−1^ attributed to zeolitic waters present in the structure of the palygorskite [84], band 1193 cm^−1^ attributed to the stretching of the Si-O-Si bond and bands below 974 cm^−1^ attributed to the vibrational modes of the octahedral cations located in the structure of the palygorskite [85].

In the acid-functionalized sample, the vibrational bands located at 3546 cm^−1^ attributed to the O-H stretch of the octahedral cations on the edges of the palygorskite channels decrease in intensity, due to the severity of the acid treatment. The same behavior can be observed at 1645 cm^−1^, attributed to the deformation of O-H groups in the water, and also in vibrational bands below 974 cm^−1^, attributed leaching to octahedral cations at the edge of the structure. There was also a narrowing of the vibrational band corresponding to 974 cm^−1^, which can be attributed to the stoichiometric increase in silica. In addition, an increase in vibrational intensity of 800 cm^−1^ corresponds to the formation of silanol groups, which is fundamental in the adsorptive process [26,40,53,86,87,88,89].

In the organo-functionalized sample, in addition to the characteristic bands of the raw sample, the presence of new bands in the spectrum (2919 cm^−1^ and 2842 cm^−1^) were also observed, referring to the vibrations of asymmetric and symmetric axial deformation of the CH_3_ groups. At 1460 cm^−1^, referring to the flexion vibrations of the CH_3_ groups, showing the accommodation of the surfactant [90,91]. The drug was observed a band at 3297 cm^−1^ related to hydroxyl vibration. The band observed between 1548–1383 cm^−1^ is attributed to the elongation of the carboxyl ion. The 1310 cm^−1^ band is attributed to the stretching to the C-N group and in 750 cm^−1^ band can be attributed to the C-Cl bond [92,93,94,95,96]. In the acid-functionalized sample after adsorption, there was a slight reduction in the vibrational band (1193 cm^−1^) corresponding to the Si-O-Si group, evidencing a possible adsorptive interaction between the drug and the respective group mentioned. New bands 1548 and 1383 cm^−1^ were also observed, in relation to the functionalized samples, probably attributed to the asymmetrical and symmetrical elongation of the carboxyl group, respectively, which can be attributed to the interaction by surface phenomenon under the active sites and non-electrostatic interactions between the carboxylate group of the drug and the surface of functionalized clays.

The change in the characteristics of raw palygorskite clays to clays with a more significant number of active sites favored interactions with the respective drug, either by surface chemistry with the formation of silanol groups by acid functionalization, where the adsorption process is probably due to electrostatic attraction between silicate ions (SiO_4_^−^) present in the clay structure and sodium of the SD carboxylate group. It is also likely due to the non-electrostatic attraction between the alkyl groups of the quaternary (cationic) ammonium surfactant present in the structure present in the organo-functionalized and the carboxylate (anionic) group of the study drug, or aromatic ring of the drug.

### 3.7. Comparison with Other Adsorbents of Sodium Diclofenac

**Figure 11:**
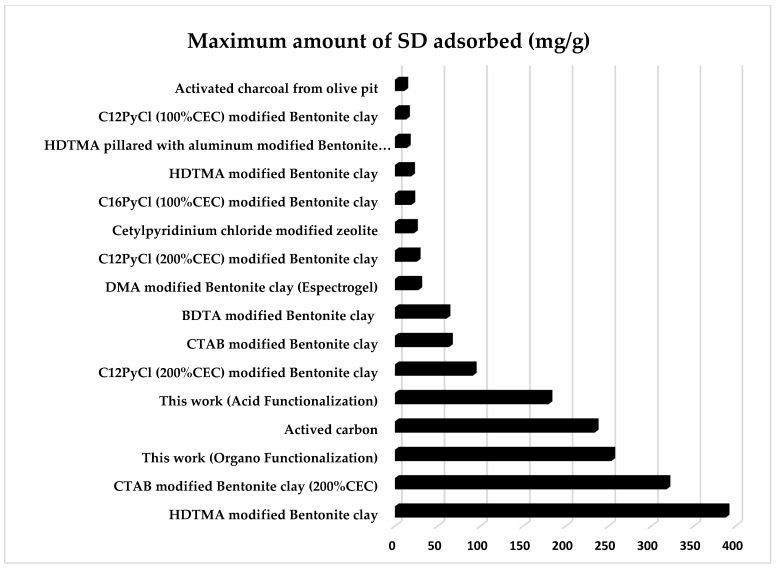
Comparison of the adsorption capacity of different adsorbents for the drug SD.

Figure 11 compares experimental data from this work with other works previously published [9,18,27,28,29,32,33,97]. From the literature, activated carbon presented the maximum amount of adsorbed SD equal to 233.9 mg/g [98], organo-functionalized zeolites that showed a maximum amount of adsorbed SD equal to 22.32 mg/g [99]. For the clays, SD adsorption studies were found only for bentonite clays. Among them the bentonite clays stand out, modified with HDTMA [32] and CTAB (200% CEC) [33], which adsorbed to more significant amounts of SD, 388 mg/g and 318g/g, respectively. In this work, the maximum amounts of adsorbed SD were 179.88 mg/g and 253.34 mg/g for acid and organo-functionalized palygorskite clay, respectively. Such experimental results indicate that the acidic or organic functionalized Brazilian palygorskite has a formidable adsorption capacity and can be an additional alternative to conventional wastewater treatment.

## 4. Conclusions

The adsorption of the drug sodium diclofenac on functionalized palygorskites has been formidably investigated. The functionalizations promoted relevant changes in the structure of the palygorskite, favoring its adsorptive potential. The results obtained by the experimental design show that the variables mass of the adsorbent, concentration of the solution are factors of great influence on the drug adsorption process and the pH had a low influence. In the kinetic study, the pseudo-second-order equation better described the adsorption process for both functionalized samples, indicating that the adsorption behavior occurs by the phenomenon of chemisorption as a probable mechanism. Regarding the adsorption isotherms, the experimental data were more suitable for the Langmuir models, indicating the existence of homogeneous adsorption sites in monolayers on the surface of the palygorskite, with a maximum drug adsorption capacity of 179.88 mg/g and 253.34 mg/g in the clays acid and organo-functionalized, respectively. In general, the studied clays proved to be suitable adsorbents for removal of sodium diclofenac present in wastewater.

## Figures and Tables

**Table 1:** Decoded and real values of the variables independent of the experimental design in the adsorption of sodium diclofenac.

Design I	Level		
Independent variables	−1	0	+1
Mass of acid functionalized (g)	0.2	0.3	0.4
Concentration of the solution (mg·L^−1^)	20	30	40
solution pH	6	8.5	11
Contact time (h)	4	6	8
Design II	Level		
Independent variables	−1	0	+1
Mass of organo-functionalized (g)	−1	0	1
Concentration of the solution (mg·L^−1^)	20	30	40
solution pH	6	8.5	11
Contact time (h)	4	6	8

**Table 2:** Chemical composition (wt%) of the raw and functionalized samples.

Oxides	Raw	Acid	Organic
SiO_2_	61.9	76.0	62.0
Al_2_O_3_	18.1	11.1	18.0
Fe_2_O_3_	8.6	5.9	8.1
MgO	6.5	5.0	6.5
K_2_O	2.8	0.6	2.7
TiO_2_	0.7	0.7	0.7
Cl	-	-	0.8
CaO	0.5	-	0.4
Others	0.9	0.7	0.8

**Table 3:** Surface areas of raw and functionalized samples.

Sample	Specific Surface Area (m^2^/g)
Raw	87.233
Acid	142.447
Organic	26.969

**Table 4:** Values of the independent variables and response variables Q_t_ of the factorial design of the functionalized palygorskite clays.

Experimental Conditions					Acid			Organic		
Exp.	Mass (g)	Initial Conc. (mg·L^−1^)	pH	Time (h)	Final Conc. (mg·L^−1^)	Q_t_ (mg g^−1^)	Rem (%)	Final Conc. (mg·L^−1^).	Q_t_ (mg g^−1^)	Rem (%)
1	0.2	20	6	4	2.031	1.7969	98.25	2.015	1.7985	89.93
2	0.4	20	6	4	2.808	0.8596	85.96	2.931	0.8534	85.35
3	0.2	40	6	4	2.32	3.768	94.20	5.8	3.4200	85.50
4	0.4	40	6	4	4.009	1.7996	89.98	6.066	1.6967	84.84
5	0.2	20	11	4	4.392	1.5608	78.04	8.698	1.1302	56.51
6	0.4	20	11	4	6.363	0.6819	68.19	8.384	0.5808	58.08
7	0.2	40	11	4	6.661	3.3339	83.35	10.074	2.9926	74.82
8	0.4	40	11	4	6.477	1.6762	83.81	18.827	1.0587	52.94
9	0.2	20	6	8	2.295	1.7705	88.53	2.342	1.7658	88.29
10	0.4	20	6	8	4.388	0.7806	78.06	3.065	0.8467	84.68
11	0.2	40	6	8	5.464	3.4536	86.34	6.251	3.3749	84.38
12	0.4	40	6	8	4.52	1.7740	88.70	5.959	1.7020	85.10
13	0.2	20	11	8	4.378	1.5622	78.11	9.791	1.0209	51.05
14	0.4	20	11	8	6.445	0.6778	67.78	8.262	0.5869	58.69
15	0.2	40	11	8	6.827	3.3173	82.94	10.366	2.9634	74.09
16	0.4	40	11	8	7.039	1.6481	82.41	19.343	1.0329	51.64
17	0.3	30	8.5	6	4.332	1.7112	85.56	4.569	1.6954	84.77
18	0.3	30	8.5	6	3.99	1.7340	86.70	4.225	1.7183	85.92
19	0.3	30	8.5	6	4.002	1.7332	86.66	4.523	1.6985	84.92

**Table 5:** ANOVA and coefficient of determination of the factorial design of acid-functionalized sample with the variable (Q_t_).

Variation Source	SQ	DF	MS	F_cal_	F_tab_	R^2^
Regression	15.8543	11	1.4413	318.53	3.605	0.9949
Residual	0.0317	7	0.0045			
Total	15.8860	18				

SQ—Sum of squares, DF—Degrees of freedom, MS—Mean square, R^2^—Determination coefficient.

**Table 6:** ANOVA and coefficient of determination of the factorial design of organo-functionalized sample with the variable (Q_t_).

Variation Source	SQ	DF	QM	F_cal_	F_tab_	R^2^
Regression	19.0551	11	1.73228	282.99	3.605	0.9942
Residual	0.0428	7	0.00612			
Total	19.0979	18				

**Table 7:** Quantification of effects of the independent variables on the response variable Q_t_ of the factorial design of palygorskite clay. (**a**) Acid and (**b**) organo-functionalized samples.

**(a)**			
**Terms**	**Effects**	**Regression Coefficient**	***p*-Value**
Mass	−1.3332	−0.6666	0
Concentration	1.3939	0.697	0
pH	−0.1931	−0.0965	0.001
Time	−0.0616	−0.0308	0.11
Mass and Conc. interation	−0.4161	−0.2081	0
Mass and pH interation	0.0606	0.0303	0.115
Mass and time interation	0.0274	0.0137	0.442
Conc. and pH interation	−0.0207	−0.0104	0.557
Conc. and time interation	−0.0401	−0.0201	0.272
pH and time interation	0.0497	0.0249	0.183
**(b)**			
**Terms**	**Effects**	**Regression Coefficient**	***p*-Value**
Mass	−1.2624	−0.6322	0
Concentration	1.5246	0.7623	0
pH	−0.5106	−0.2553	0
Time	−0.0305	−0.0153	0.461
Mass and Conc. interation	−0.7173	−0.3591	0
Mass and pH interation	0.0507	0.0253	0.236
Mass and time interation	0.0253	0.0126	0.539
Conc. and pH interation	−0.2431	−0.1216	0
Conc. and time interation	−0.0148	−0.0074	0.716
pH and time interation	−0.0108	−0.0054	0.791

**Table 8:** Kinetic parameters and ANOVA obtained experimental data of SD adsorption in acid-functionalized sample fitting to the models of pseudo-first-order, pseudo-second-order and Elovich.

**Pseudo-First Order Model**							
**Parameters**	**Variation Source**	**SQ**	**DF**	**MS**	**F_cal_**	**Error**	**R^2^ (adj)**
K_1_ = 0.0264 min^−1^	Regression	9410.097	2	4705.048	738.72	1.24	0.96
Qef = 36.0295 mg/g	Residual	63.690	10	6.36913			
	Total	9473.788	12				
**Pseudo-Second Order Model**							
**Parameters**	**Variation Source**	**SQ**	**DF**	**MS**	**F_cal_**	**Error**	**R^2^ (adj)**
K_2_ = 0.000729 g·min^−1^·min^−1^	Regression	9421.694	2	4710.847	904.31	1.96	0.97
Qef = 42.424 mg/g	Residual	52.093	10	5.209			
	Total	9473.788	12				
**Elovich Model**							
**Parameters**	**Variation Source**	**SQ**	**DF**	**MS**	**F_cal_**	**Error**	**R^2^ (adj)**
α = 5.27131 mg/g·min	Regression	9404.827	2	4702.414	681.9	1.51	0.95
β = 0.04598 g/mg	Residual	68.960	10	6.89605			
	Total	9473.788	12				

**Table 9:** Kinetic parameters and ANOVA obtained experimental data of SD adsorption in organo-functionalized sample fitting to the pseudo-first-order, pseudo-second-order and Elovich models.

**Pseudo-First Order Model**							
**Parameters**	**Variation Source**	**SQ**	**DF**	**MS**	**F_cal_**	**Error**	**R^2^ (adj)**
K_1_ = 0.27775 min^−1^	Regression	16,561.380	2	8280.69	4933.21	0.41	0.985
Qef = 38.09673 mg/g	Residual	18.464	11	1.67856			
	Total	16,579.842	13				
**Pseudo-Second Order Model**							
**Parameters**	**Variation Source**	**SQ**	**DF**	**MS**	**F_cal_**	**Error**	**R^2^ (adj)**
K_2_ = 0.01722 g·min^−1^·min^−1^	Regression	16,573.664	2	8286.83	14,753.23	0.28	0.995
Qef = 39. 06836 mg/g	Residual	6.178	11	0.56			
	Total	16,579.842	13				
**Elovich Model**							
**Parameters**	**Variation Source**	**SQ**	**DF**	**MS**	**F_cal_**	**Error**	**R^2^ (adj)**
α = 5.10^7^ mg/g·min	Regression	16,556.29	2	8278.146	3866.58	1.84	0.981
β = 0.23802 g/mg	Residual	23.55	11	2.14			
	Total	16,579.84	13				

**Table 10:** Parameters and ANOVA obtained experimental data of SD adsorption in acid-functionalized sample fitting to the isotherm models of Langmuir, Freundlich and Temkin.

**Langmuir Model**							
**Parameters**	**Variation Source**	**SQ**	**DF**	**MS**	**F_cal_**	**Error**	**R^2^ (adj)**
K_L_ = 0.00545	Regression	53,210.65	2	26,605.33	655.69	0.91	0.97795
Q_max_ = 179.87849	Residual	486.91	12	40.5761			
	Total	53,697.57	14				
**Freundlich Model**							
**Parameters**	**Variation Source**	**SQ**	**DF**	**MS**	**F_cal_**	**Error**	**R^2^ (adj)**
K_F_ = 3.40006	Regression	52,332.94	2	26,166.47	230.096	1.07	0.93891
N = 1.63677	Residual	1364.63	12	113.72			
	Total	53,697.57	14				
**Temkin Model**							
**Parameters**	**Variation Source**	**SQ**	**DF**	**MS**	**F_cal_**	**Error**	**R^2^ (adj)**
αt = 0.08844	Regression	20,587.27	1	20,587.27	164.793	0.65	0.93212
βt = 0.03178	Residual	1499.134	12	124.927			
	Total	22,086.41	13				

**Table 11:** Parameters and ANOVA obtained experimental data of SD adsorption in organo-functionalized sample fitting to the isotherm models of Langmuir, Freundlich and Temkin.

**Langmuir Model**							
**Parameters**	**Variation Source**	**SQ**	**DF**	**MS**	**F_cal_**	**Error**	**R^2^ (adj)**
K_L_ = 0.00371	Regression	71,632.444	2	35,816.222	1373.097	0.49	0.99
Q_max_ = 253.34452	Residual	313.011	12	26.084			
	Total	71,945.455	14				
**Freundlich Model**							
**Parameters**	**Variation Source**	**SQ**	**DF**	**MS**	**F_cal_**	**Error**	**R^2^ (adj)**
K_F_ = 2.78045	Regression	70,937.93	2	35,468.97	422.45	0.71	0.965
N = 1.47776	Residual	1007.52	12	83.96			
	Total	71,945.455	14				
**Temkin Model**							
**Parameters**	**Variation Source**	**SQ**	**DF**	**MS**	**F_cal_**	**Error**	**R^2^ (adj)**
αt = 0.08236	Regression	28,839.644	1	28,839.644	119.194	0.65	0.908
βt = 0.02685	Residual	2903.45	12	241.95			
	Total	31,743.095	13				

## Data Availability

Not applicable.
